# Oxidized Cell‐Free Hemoglobin Induces Mitochondrial Dysfunction by Activation of the Mitochondrial Permeability Transition Pore in the Pulmonary Microvasculature

**DOI:** 10.1111/micc.70012

**Published:** 2025-05-20

**Authors:** Kyle J. Riedmann, Jamie E. Meegan, Aqeela Afzal, Yatzil Cervantes‐Cruz, Sarah Obeidalla, Avery M. Bogart, Lorraine B. Ware, Ciara M. Shaver, Julie A. Bastarache

**Affiliations:** ^1^ Department of Cell and Developmental Biology Vanderbilt University Nashville Tennessee USA; ^2^ Division of Allergy, Pulmonary, and Critical Care Medicine Vanderbilt University Medical Center Nashville Tennessee USA; ^3^ Department of Neurosurgery Vanderbilt University Medical Center Nashville Tennessee USA; ^4^ Department of Pathology, Microbiology, and Immunology Vanderbilt University Nashville Tennessee USA

**Keywords:** cell‐free hemoglobin, mitochondrial dysfunction, mitochondrial permeability transition pore, mtDNA

## Abstract

**Objective:**

Cell‐free hemoglobin (CFH) is released into the circulation during sepsis where it can redox cycle from the ferrous 2+ to ferric 3+ and disrupt endothelial function, but the mechanisms of CF‐mediated endothelial dysfunction are unknown. We hypothesized that oxidized CFH induces mitochondrial dysfunction via the mitochondrial permeability transition pore (mPTP) in pulmonary endothelial cells, leading to the release of mitochondrial DNA (mtDNA).

**Methods:**

Human lung microvascular endothelial cells were treated with CFH2+/CFH3+. We measured mitochondrial mPTP activation (flow cytometry), network and mass (immunostaining), structure (electron microscopy), mtDNA release (PCR), and oxygen consumption rate (OCR; Seahorse). Plasma from critically ill patients and conditioned cell media were quantified for mtDNA and CFH.

**Results:**

CFH3+ disrupted the mitochondrial network, activated the mPTP (1434 (874–1642) vs. 2302 (1729–2654) mean fluorescent intensity, *p* = 0.02), increased the spare respiratory capacity (30.61 (29.36–37.78) vs. 7.83 (3.715–10.63) OCR, *p* = 0.004), and caused the release of mtDNA. CFH was associated with circulating mtDNA (*R* ^2^ = 0.1912, *p* = 0.0077) in plasma from critically ill patients.

**Conclusion:**

CFH3+, not CFH2+, is the primary driver of CFH‐induced lung microvascular mitochondrial dysfunction. Activation of the mPTP and the release of mtDNA are a feature of CFH3+ mediated injury.

## 1 Introduction

Sepsis results from a dysregulated immune response to an infection [1] and remains a critical problem accounting for ~20% of global deaths [2]. The highly inflammatory environment during sepsis causes systemic damage to organs and the vascular system [3]. In the lungs, this manifests as pulmonary microvasculature disruption leading to alveolar edema, leukocyte migration, and vasodilation [3, 4]. The pulmonary microvascular endothelium is critical for maintaining tissue homeostasis and barrier function of the alveolar‐capillary barrier [5]. Endothelial dysfunction results in part from circulating inflammatory mediators, microbial products, and damage‐associated molecular patterns (DAMPs) [6, 7, 8]. Cell‐free hemoglobin (CFH) is a DAMP released from damaged red blood cells (RBC) and is elevated in ~80% of critically ill patients with sepsis [9]. CFH is a known driver of pulmonary vascular dysfunction via several mechanisms including signaling through toll‐like receptor 4 (TLR4), activation of the NLR family pyrin domain‐containing protein 3 (NLRP3) inflammasome, and generation of reactive oxygen species (ROS) [10, 11, 12, 13, 14, 15]. The significant increase in ROS caused by CFH suggests that downstream ROS‐sensitive mitochondrial processes may be implicated in mediating CFH‐induced endothelial dysfunction.

Circulating CFH can redox cycle between the ferrous (CFH2+) and ferric (CFH3+, methemoglobin) iron forms, generating ROS [15]. The effects of CFH on the endothelium include increased barrier permeability, leukocyte adhesion, and oxidative damage [10, 16, 17, 18]. Studies evaluating these effects on the endothelium have focused on the previously mentioned pathways, but perturbations to mitochondrial function have also been reported. In alveolar epithelial cells, CFH3+ induces translocation of heme oxygenase 1 (HO‐1) to the mitochondria and causes partial depolarization of the mitochondrial network [19]. Disruption of mitochondrial function in endothelial cells has been linked to impaired proliferation and nitric oxide (NO) signaling [20]. The precise mitochondrial dynamics that occur during CFH‐induced endothelial dysfunction remain unclear. Analysis of these changes to mitochondrial function by CFH offers insight into molecular mechanisms underlying CFH‐induced endothelial dysfunction and additionally downstream how mitochondrial products are released in response to mitochondrial dysfunction.

This study aims to evaluate the differential effects of CFH2+ and CFH3+ on mitochondrial structure and function in the pulmonary microvascular endothelium. We hypothesize that CFH3+ induces mitochondrial dysfunction in endothelial cells as evidenced by changes in mitochondrial mass, polarization, morphology, and oxidative respiration, resulting in activation of the mPTP and release of mtDNA.

## 2 Methods and Materials

### 2.1 Cells and Culture Conditions

Primary human lung microvascular endothelial cells (HLMVECs) were purchased from PromoCell (C‐12281; Heidelberg, Germany) and cultured in Endothelial Cell Growth Media MV2 (C‐22121; PromoCell). Cells were grown to confluence on tissue culture plates coated with Attachment Factor Protein (S006100; Gibco) in a humidified 5% CO_2_ incubator at 37°C. Hemoglobin was isolated, purified, and oxidized via UV irradiation for 3 h with a 10 min break every 30 min to prevent overheating as previously described [21]. Cells were treated with purified cell‐free hemoglobin (CFH) diluted in PBS at 1.0 mg/mL or PBS vehicle control at timepoints ranging from 1 to 24 h [17, 18, 22, 23, 24]. In some experiments, cells were pre‐treated for 1 h with haptoglobin (Hp; gift from CSL Behring) at a 2:1 M ratio (15.5 μM, human plasma purified). Hp is the endogenous CFH scavenger that reliably ameliorates the effects of CFH and is used as a negative control. Hp works through structural stabilization and prevention of oxidative reactions when bound to CFH [25]. All experiments were conducted with HLMVECs from at least one male and one female donor, up to three male and two female donors depending on availability, and HLMVECs were used below passage 7 at full confluency.

### 2.2 Widefield Fluorescent Microscopy

Cells were plated and cultured on black 96‐well plates coated with Attachment Factor (S006100; Gibco) and allowed to reach confluency. Cells were treated with vehicle, CFH2+, CFH3+ (1.0 mg/mL), or paraquat (5 mM) for 6 h prior to staining with CellROX Deep Red (5 uM) or MitoTracker Green (100 nM) for 30 min at 37°C. Hp pretreatment for 1 h was performed for CellROX experiments. Cells were fixed and DNA counterstained as above. Plates were imaged on Lionheart FX (BioTek Instruments Inc., Winooski, VT) with 2 × 2 montage at ×4 magnification using the DAPI and FITC or RFP channels for MitoTracker Green or CellROX Deep Red, respectively. Analysis was performed on a secondary mask of Hoechst (500 ng/mL) stained cells to quantify the object mean fluorescence intensity (MFI) to generate a cell number independent value.

### 2.3 Mitochondrial ROS, Membrane Polarization, and mPTP Activation

HLMVECs were grown in 6‐well plates until confluency before treatment with Vehicle (PBS, 1:10), CFH2+, or CFH3+ (1.0 mg/mL) for either 6 or 24 h depending on the assay. For the flow assays, HLMVECs were washed once with PBS, incubated with TrypLE Express Enzyme (12 604 013; Gibco) at 37C for 3–5 min for detachment, collected into prewarmed complete media prior to centrifugation at 500× *g* for 5 min to pellet the cells. For the mPTP assay, cell pellets were incubated with the combination of calcein‐AM and cobalt chloride included in the MitoProbe Transition Pore Assay Kit (M34153; Invitrogen, Carlsbad, CA) in HBSS (21‐020CV; Corning, Corning, NY) for 30 min in a 37°C incubator. Cells were analyzed using a LSRFortessa (BD, Franklin Lakes, NJ) in the FITC channel to obtain the MFI. For the MitoSOX Deep Red experiments, cells were stained (5 uM) in 6‐well plates after treatment for 30 min at 37°C before harvesting as described above. Cells were analyzed on an LSRFortessa using the PE filter set. Both the mPTP and MitoSOX assays required minimal compensation with unstained cells and the single channel for the respective dye. For the JC10 assay, the cells were treated and harvested as described above before being resuspended in 1× JC10 in assay buffer (ab112133; Abcam, Cambridge, MA) and incubated at 37°C for 30 min prior to analysis on an LSRFortessa using the FITC and PE filter sets to obtain the PE:FITC ratio. Spectral compensation was performed on unstained, JC10 stained cells (PE channel), and JC10 stained cells treated with FCCP (2.5 uM, FITC channel). FCCP optimization showed the highest FITC:PE ratio was 2.5 μM for 30 min at 37°C (Figure S4). Data from all flow cytometry experiments were analyzed using FlowJo (FlowJo, Ashland, OR; Version 10.10.0).

### 2.4 Mitochondrial Morphology by Electron Microscopy

HLMVECs were grown to confluency in 100 mm plates and treated with vehicle, CFH2+, or CFH3+ for 6 h. After washing with HBSS, the cells were fixed with 2.5% glutaraldehyde and 1% paraformaldehyde in 0.1 M cacodylate buffer for 60 min at room temperature. Cell pellets were post‐fixed sequentially in 1% tannic acid, 1% osmium tetroxide, and en bloc stained in 1% uranyl acetate [26]. Samples were dehydrated in a graded ethanol series and infiltrated with Quetol 651 low viscosity Spurr's Resin using propylene oxide as the transition solvent. The resin was polymerized at 60°C for 48 h, sectioned using a Leica Enuity at a nominal thickness of 70 nm, and collected onto 300 mesh Ni grids. Sections were stained with 2% uranyl acetate and lead citrate. Samples were imaged either on a ThermoFisher Scientific Tecnai T12 operating at 100 keV using an AMT nanosprint 5 CMOS camera or a JEOL 2100 Plus operating at 200 keV using an AMT nanosprint 15mkII CMOS camera. Images were obtained at 6500× and 6000× magnification, respectively. Mitochondrial morphology parameters were measured with Fiji, an open‐source application used for image analysis, on individual mitochondrion by a blinded reviewer [27]. At least 100 mitochondria were measured per group by selection of the mitochondrion using the circular selection tool before taking measurements of the mean intensity (mean electron density), roundness, and circularity. Results are from two male donors with > 10 fields of view and > 100 mitochondria per group.

### 2.5 Seahorse Mito Stress Test

Metabolic assays were performed using the Seahorse Analyzer (Agilent) and the Mito Stress Test (103015–100, Agilent) drug packs. HLMVECs were plated at confluency on coated XFe96 (103794–100, Agilent) plates and allowed to form a monolayer. Cells were treated with Vehicle, CFH2+, or CFH3+ (1.0 mg/mL) for 6 h. The Mito Stress Test was performed according to the manufacturer's instructions. Optimized concentrations of the drugs were previously found to be 1.5 uM oligomycin, 1 uM FCCP, and 0.5 uM each of rotenone and antimycin A. The *media* used was XF DMEM (103680–100, Agilent) with 1 mM pyruvate, 2 mM glutamine, and 10 mM glucose. Oxygen consumption rates were normalized to cell count per well. Results are from six replicates averaged together for the displayed data points. Data are from five different donors, with some donors repeated twice.

### 2.6 Confocal Immunofluorescence

For confocal microscopy, poly‐L‐lysine coated coverslips (GG‐12‐1.5‐PLL; Neuvitro, Camas, WA) were coated with Attachment Factor for 30 min at 37°C and placed inside 12‐well plates for confluent seeding of HLMVECs in complete MV2 media. After monolayer formation, cells were treated with vehicle, CFH2+ or CFH3+ (1.0 mg/mL) for 6 h. Paraquat (2 mM), a direct ROS inducer, and antimycin A (5 uM), a complex III inhibitor, were used as positive controls for ROS formation and mitochondrial function disruption, respectively. Treated HLMVECs were rinsed with PBS twice before fixation with 4% paraformaldehyde in PBS for 10 min in the dark at room temperature. Cells were permeabilized after washing with PBS using 0.1% Triton X‐100 in PBS for 10 min at room temperature. Coverslips were washed 3× with PBS before blocking in 10% normal donkey serum in PBS for 1 h at room temperature. Coverslips were incubated with primary antibody overnight in blocking buffer at 4°C. Antibodies and dyes used were rabbit anti‐vascular‐endothelial cadherin [D87F2] (1:400, 2500S; Cell Signaling Tech) for junction labeling and cell segmentation, MitoTracker Orange (150 nM, M7510; Invitrogen) for mitochondrial network analysis, and Hoechst (500 ng/mL, H1398; Invitrogen) as a DNA counterstain. After primary incubation, coverslips were washed with PBS three times for 5 min/wash, and incubated with the secondary antibodies donkey anti‐rabbit Alexa Fluor 488 (1:1000, A‐21206; Invitrogen) or donkey anti‐mouse Alexa Fluor 647 (1:1000, A‐31571; Invitrogen) for 1 h at room temperature in the dark. After 3X PBS washes, the coverslips were mounted on glass slides with ProLong Diamond (P36961, Invitrogen) and allowed to cure before imaging. Slides were imaged on the Zeiss LS780 with a 63X oil‐immersion objective. Quantification of mitochondrial network changes was done via Fiji using the mitochondrial network analysis (MiNA) plugin as previously described [27, 28]. Briefly, the mitochondria channel images (MitoTracker Orange) were preprocessed using a median filter, top hat filter, and contrast limited AHE (CLAHE) in that order before ridge detection for network analysis [28].

### 2.7 Real‐Time Quantitative PCR

Total RNA from treated HLMVEC was extracted using Qiagen RNeasy Mini Kit (74104; Qiagen, Hilden, Germany) per the manufacturer's instructions. Total RNA of 1 μg was used per 20 μL of iScript cDNA Synthesis Kit (1708891; Bio‐Rad, Hercules, CA). Bio‐Rad Prime PCR primers and probes specific to mt‐ND4L (qHsaCEP0055828), mt‐CYB (qHsaCEP0055614), and GAPDH (qHsaCEP0041396) were used to analyze extracellular DNA with qPCR. Qualitative PCR amplifications were performed in a QuantStudio 3 (A28567, Applied Biosystems) thermocycler using TaqMan Advanced Fast Master Mix (4444557; Applied Biosystems) with the following program: (20 s at 95°C; 40 cycles of 1 s at 95°C then 20 s at 60°C). DNA from conditioned media, after centrifugation to remove debris or detached cells, of HLMVECs treated for 24 h were purified using Qiagen Blood and Tissue kit (69506, Qiagen) per the manufacturer's instructions. Isolated DNA was quantified for mtDNA using primers and probes specific to mitochondrially encoded ND4L (mt‐ND4L).

### 2.8 Patient Plasma

Plasma was previously collected from a prospective cohort of critically ill patients at Vanderbilt University Medical Center (written informed consent was obtained and approved by Vanderbilt Institutional Review Board, Protocol No. 051065). The Validating Acute Lung Injury biomarkers for Diagnosis (VALID) study cohort has been extensively characterized previously for biomarker and risk factor studies [9, 29, 30]. Plasma was obtained at enrollment, generally ICU day 2, from patients admitted to Vanderbilt Medical, Surgical, Trauma, and Cardiovascular ICUs at high risk of developing end organ dysfunction [30]. Cell‐free hemoglobin was previously measured by ELISA (ab157707, Abcam) according to manufacturer's instructions [9].

### 2.9 Statistical Analysis

Statistical analysis was performed using GraphPad Prism v10. Comparisons between groups were made using Kruskal–Wallis one‐way ANOVA with Dunnett's multiple‐comparisons test, with an adjusted significance value set at *α* = 0.05. All graphs are shown with the median and interquartile range or violin plots unless stated otherwise.

## 3 Results

### 3.1 CFH3+ Increases Cellular and Mitochondrial ROS

**FIGURE 1:**
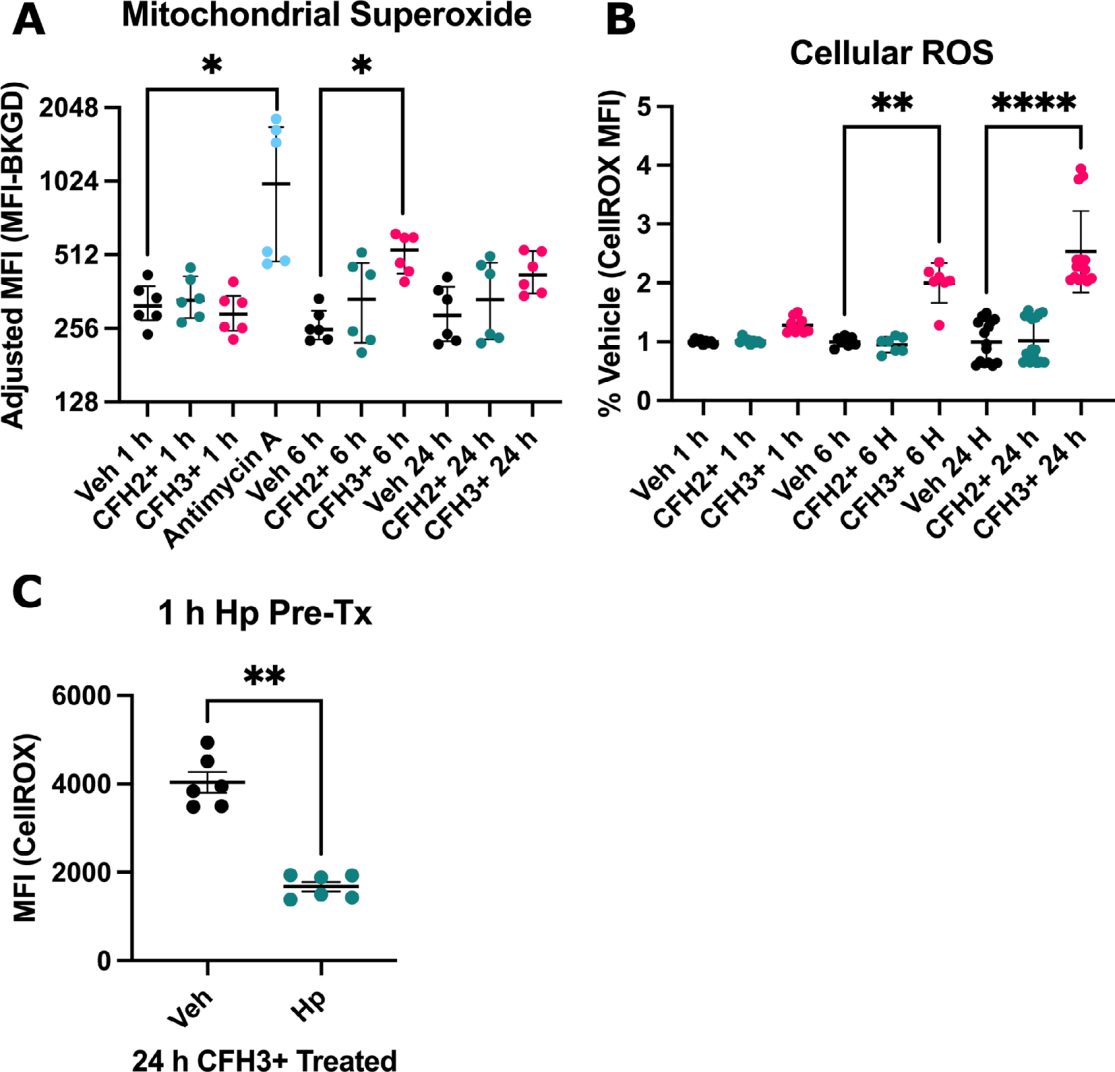
CFH3+ induces mitochondrial superoxide and cellular ROS formation. MitoSOX stained HLMVECs were treated for the indicated time points before being analyzed on a BD LSRFortessa in the PE channel. (A) Quantification by background adjusted MFI of MitoSOX staining of HLMVECs at the time points of 1, 6, and 24 h. CellROX stained HLMVECs were imaged on a BioTek Lionheart FX microscope using the Cy5 filter set (628/685). (B) Quantification of CellROX Deep Red staining at the time points of 1, 6, and 24 h. (C) Pre‐treatment with Hp for 1 h reduced CellROX staining from CFH3+ exposure at 24 h (Mann–Whitney test). (A,C) *n* = 6 from 3 donors (two male, one female), (B) *n* = 7–15 from 4 donors (two male, two female). * = *p* < 0.05, ** = *p* < 0.01 , and **** = *p* < 0.0001. Graphs show median with interquartile range.

Hemoglobin redox cycles via the iron atom present in the heme moiety. Therefore, we sought to quantify the changes in ROS production after CFH exposure. We utilized MitoSOX Red to measure mitochondrial superoxide via flow cytometry. MitoSOX MFI was significantly elevated at 6 h (536 (428–607) vs. 253 (230–302) MFI, *p* = 0.0116) after CFH3+ treatment compared to vehicle (Figure 1A). Treatment with antimycin A, an electron transport chain inhibitor, as a positive control also elevated MitoSOX MFI at 1 h post‐treatment. We also measured cellular ROS using CellROX Deep Red and found that ROS was elevated significantly at 6 and 24 h in the CFH3+ group compared to the vehicle and CFH2+ groups (Figure 1B). Pretreatment with Hp ameliorated this increase in cellular ROS at the 24 h timepoint for CFH3+ treated cells (Figure 1C). Pretreatment with antioxidants MitoQ and vitamin C did not reduce CellROX fluorescence (Figure S1). These data support that cellular ROS and superoxide are elevated during the acute 6 to 24 h time period after CFH3+ treatment.

### 3.2 Spare Respiratory Capacity Significantly Increased From CFH3+ Treatment

**FIGURE 2:**
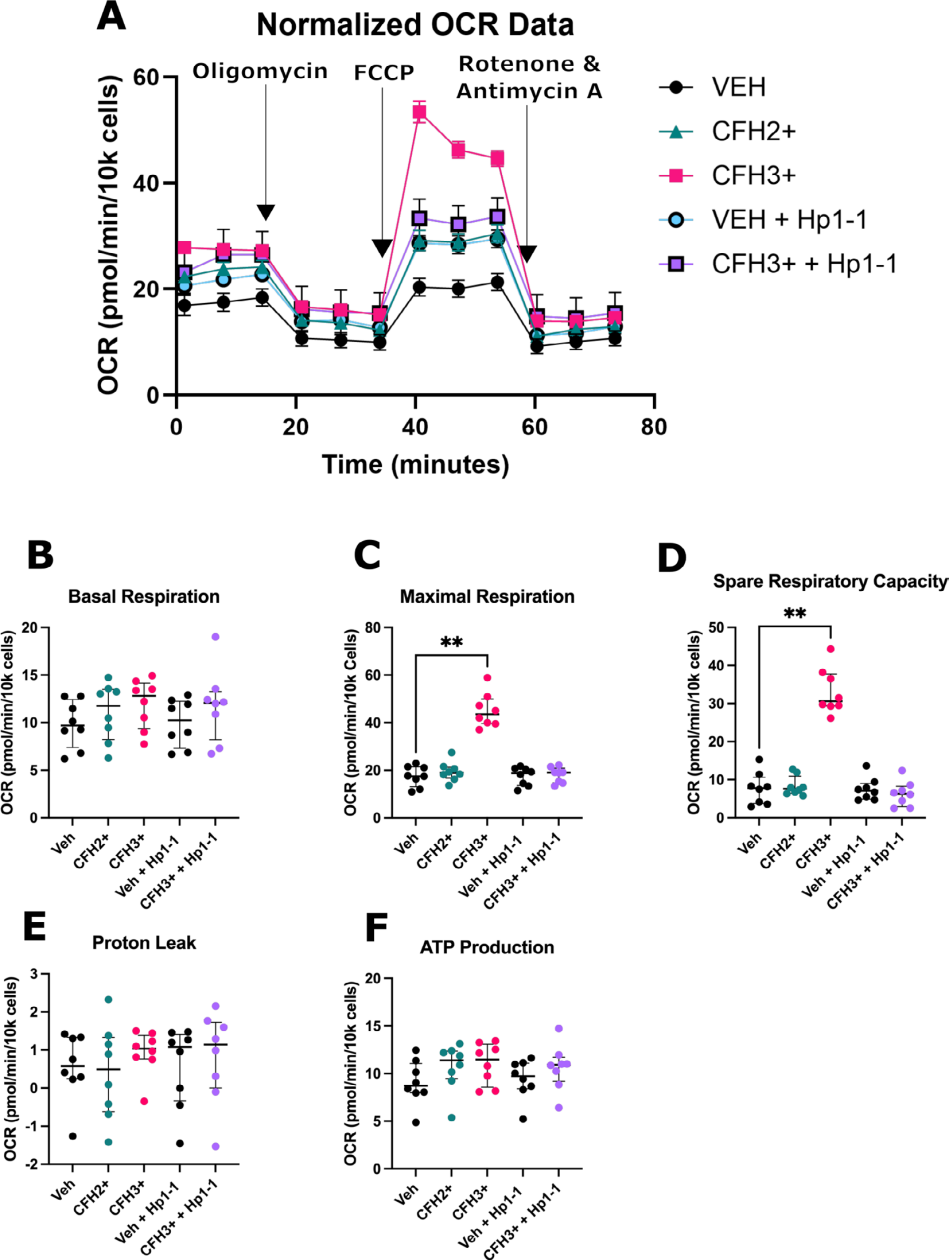
Treatment of endothelial monolayers with CFH3+ increases maximal respiration and spare respiratory capacity. Confluent HLMVEC monolayers were treated for 6 h with CFH before room temp equilibration prior to analysis on the Seahorse Analyzer using the Mito Stress Test. Hp pretreatment was for 1 h for the VEH + Hp1‐1 and CFH3++Hp1‐1 groups. (A) Normalized OCR data from donor 57F showing the change in oxygen consumption after the addition of oligomycin (~20 min), FCCP (~40 min), and rotenone and antimycin A (~60 min). Quantification of metabolic parameters of (B) basal respiration, (C) maximal respiration, (D) spare respiratory capacity, (E) proton leak, and (F) ATP production. *n* = 8 from 5 donors (three male, two female) with some donors repeated. ** = *p* < 0.01.

Elevated mitochondrial superoxide from CFH3+ treatment led us to hypothesize that oxidative respiration would be impaired from CFH treatment. To test the effects of CFH3+ on mitochondrial respiration, we utilized the Mito Stress Test assay on the Seahorse Analyzer. A representative OCR plot over time showed the changes in respiration that occur from CFH treatment (Figure 2A). A significant increase was seen in maximal respiration (43.50 [39.64–49.96] vs. 17.52 OCR [13.13–21.41], *p* = 0.001) and spare respiratory capacity (30.61 [29.36–37.78] vs. 7.83 [3.715–10.63] OCR, *p* = 0.004) when comparing CFH3+ and vehicle‐treated cells (Figure 2C,D). No significant changes in basal respiration, proton leak, and ATP production were seen (Figure 2B,E,F). The spare respiratory capacity is the cell's ability to respond to increases in energy needs and respiratory stress and has been implicated in cellular survival [31, 32]. The increases in maximal respiration and spare respiratory capacity seen here contrast with previous experiments showing dysfunction of the mitochondrial network.

### 3.3 CFH3+ Reduces the Mitochondrial Area and Circularity

**FIGURE 3:**
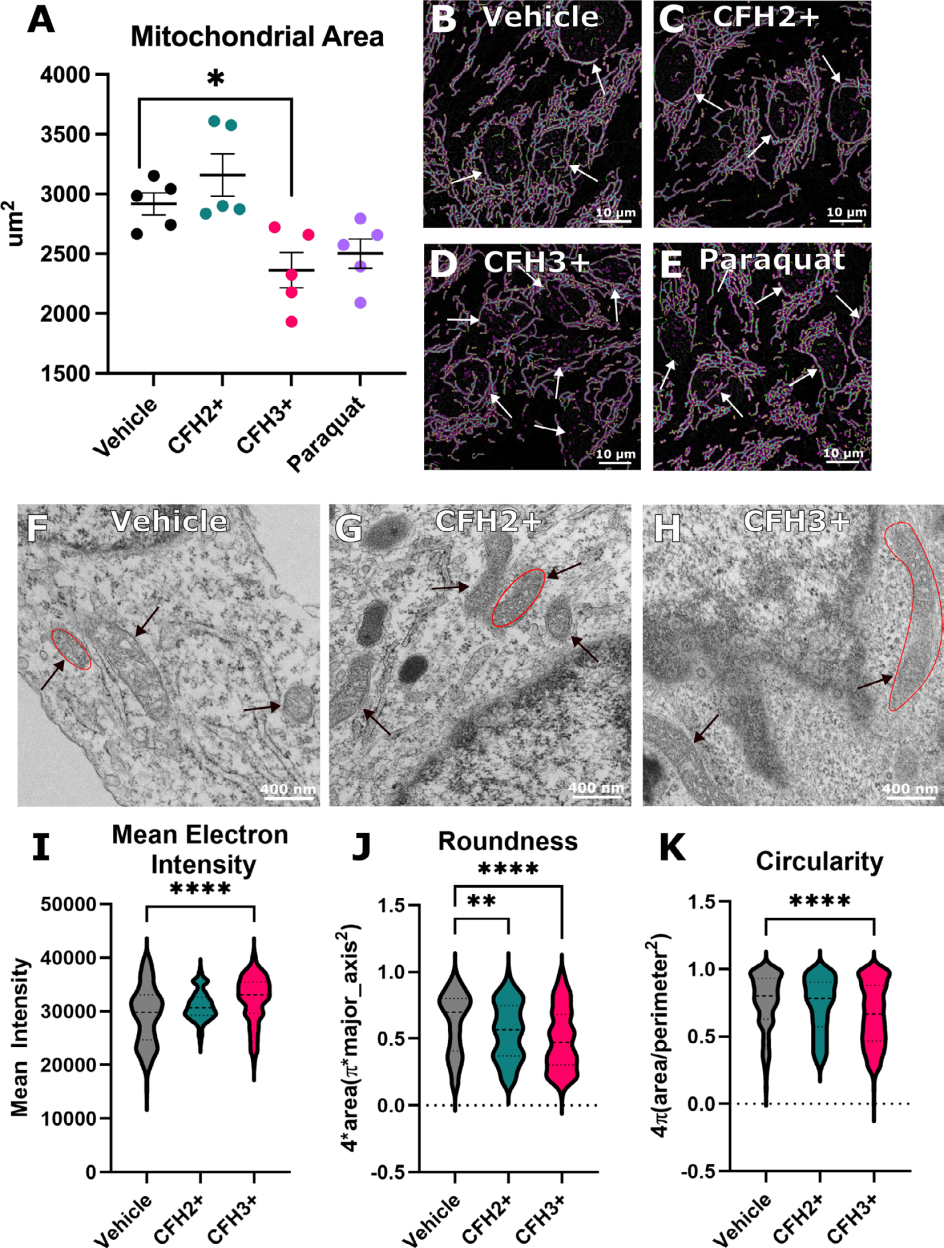
CFH3+ induces mitochondrial morphological changes. (A) Quantification of the mitochondrial footprint per field of view using the Mitochondrial Network Analysis (MiNA) ImageJ plugin. Representative images of the MiNA output for (B) vehicle, (C) CFH2+, (D) CFH3+, and (E) Paraquat treated cells with nuclei highlighted (white arrows; scale bar = 10 um). Representative TEM micrographs for (F) vehicle, (G) CFH2+, and (H) CFH3+ treated HLMVECs with circularity demonstration (red circles) and mitochondria highlighted (black arrows; scale bar = 400 nm). Quantification of mitochondrial morphology parameters of (I) mean electron density, (J) circularity (4pi (area/perimeter^2^)) and (K) aspect ratio (major axis: Minor axis). (A–E) *n* = 5, (F, vehicle) *n* = 228, (G, CFH2+) *n* = 163, (H, CFH3+) *n* = 416. * = *p* < 0.05, ** = *p* < 0.01 , and **** = *p* < 0.0001.

To test for changes in mitochondrial morphology in response to CFH we utilized confocal and transmission electron microscopy (TEM). HLMVECs stained with MitoTracker Orange were imaged via confocal microscopy, and images were processed and analyzed using the MiNA plugin for Fiji. Representative images show the mitochondrial network with the MiNA overlay that is used for parameter quantification (Figure 3B–E). Network analysis revealed that CFH3+ caused a significant 19% reduction in the total mitochondrial area compared to vehicle control (2363 vs. 2917 um^2^, *p* = 0.0485). Positive control paraquat also decreased the mitochondrial area, but it was not statistically significant (Figure 3A). Other parameters including branch length, summed branch length, network branches, and donuts were not significantly different than vehicle treated cells (Figure S2). High‐resolution TEM showed that CFH3+ causes mitochondrial morphology changes including increased mean electron density and aspect ratio and decreased circularity (Figure 3I–K). Analysis of the fission protein DRP1 did not show changes at the RNA and protein levels (Figure S3). The results show that the mitochondrial network is decreased in response to CFH3+ with individual mitochondrion becoming more oblong and less round.

### 3.4 Mitochondrial Mass Is Reduced by Treatment With CFH

**FIGURE 4:**
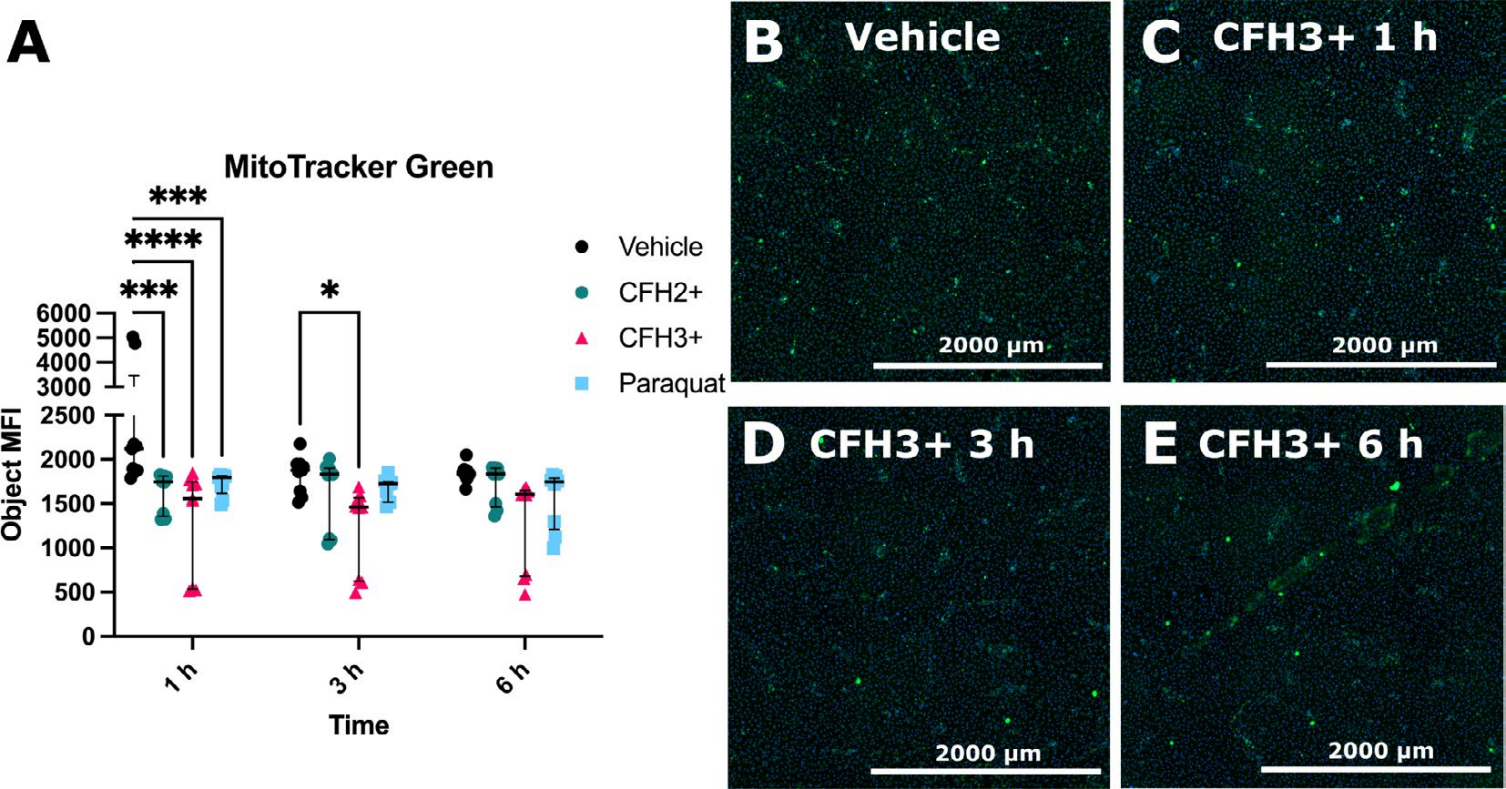
CFH3+ decreases mitochondrial mass. (A) Quantification of MitoGreen by object MFI based on the DNA counterstain. Representative images (Blue = nuclei, Green = mitochondria; scale bar = 2000 um) from HLMVECs stained with MitoTracker Green and treated with (B) vehicle and CFH3+ at the (C) 1, (D) 3, and (E) 6 h time points. *n* = 6 from 3 donors (two male, one female). * = *p* < 0.05, *** = *p* < 0.001, **** = *p* < 0.0001.

We sought to evaluate if mitochondrial mass was decreased in response to CFH treatment using MitoTracker Green staining of HLVMECs to visualize mitochondria. In CFH3+ treated cells, we observed a significant decrease in MitoTracker Green MFI when compared to control cells beginning at the 1 h timepoint (1557 [535.5–1747] vs. 2121 [1867–3473] MFI, *p* < 0.0001) and persisting until 3 h (1462 [625–1565] vs. 1877 [1606–1946] MFI, *p* = 0.03) (Figure 2A). Similar effects were seen at 6 h but were not significant (1606 [675.5–1652] vs. 1836 (1799–1880) MFI, *p* = 0.0589). The representative images demonstrate the change in MitoTracker Green staining over the time course of 1–6 h in CFH3+ treated HLMVECs (Figure 4B–E). Significant decreases in MitoTracker Green staining were also seen with both CFH2+ and paraquat, a direct ROS inducer, at the 1 h timepoint (Figure 4A). Decreases in mitochondrial mass at the earliest timepoint were seen with all groups but only CFH3+ had a reduction that persisted until the final 6 h timepoint.

### 3.5 CFH Induces Partial Depolarization of the Mitochondrial Network

**FIGURE 5:**
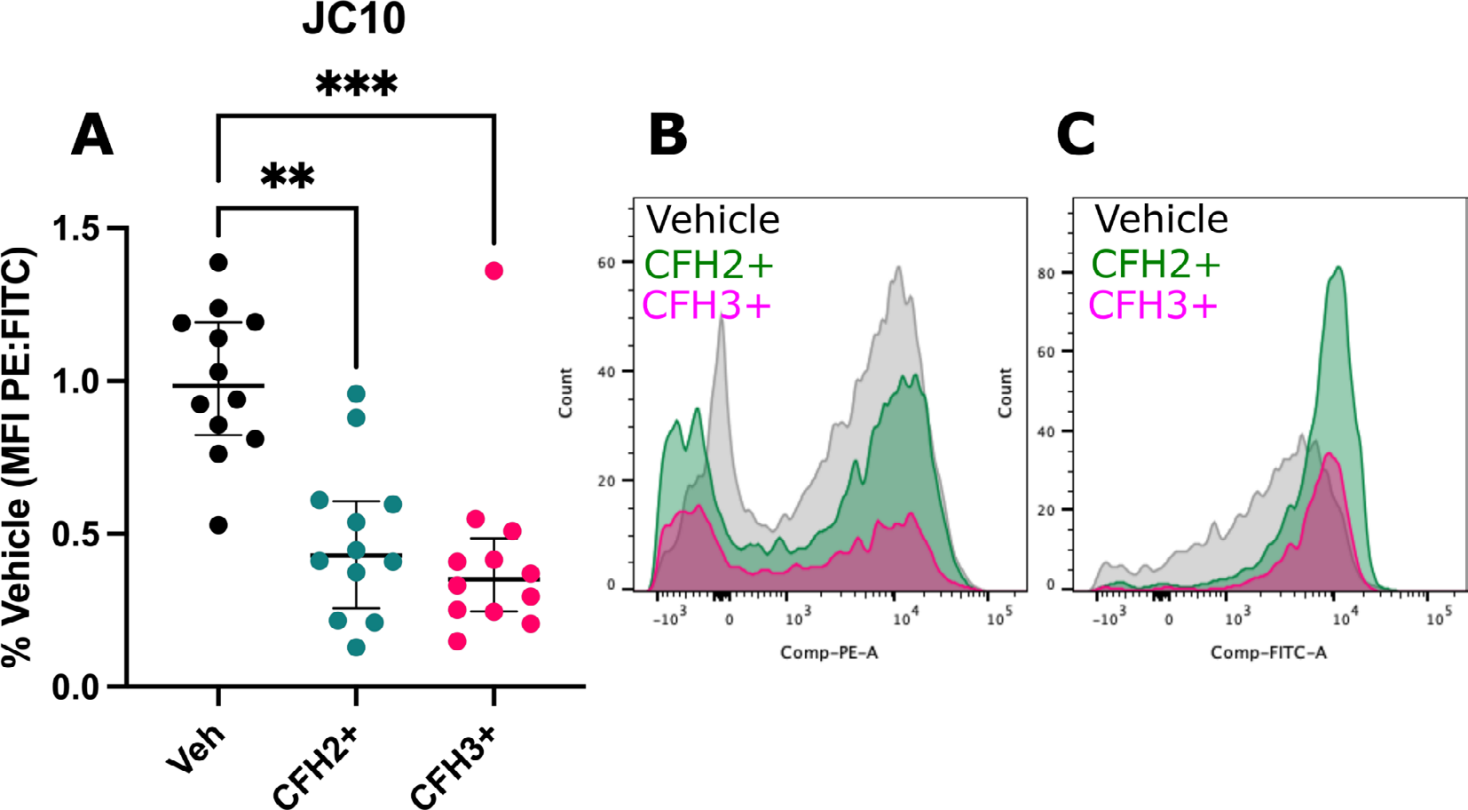
CFH causes partial depolarization of mitochondria. (A) Quantification of the shift in fluorescence by the ratio of PE:FITC channels. Representative stacked histograms of the JC10 stained cells in the (B) PE and (C) FITC filter sets. *n* = 12 from 3 donors (two male, one female). ** = *p* < 0.01 and *** = *p* < 0.001.

To identify the mechanism by which the mitochondrial network was shrinking, we evaluated changes in mitochondrial polarization. The fluorogenic probe JC10 was used due to its shift in fluorescence from green (cytosolic) to red (mitochondria) based on localization. Treatment with FCCP for compensation was optimized to 30 min with 2.5 uM FCCP (Figure S4). A reduction in the red fluorescence (PE channel) of JC10 is seen upon depolarization of mitochondria within a cell. HLMVECs treated with both CFH2+ and CFH3+ had a significant reduction of ~50% in the PE:FITC ratio at the 6 h timepoint when compared to vehicle control (Figure 5A). Representative histograms of PE and FITC fluorescence show the significant decrease in PE fluorescence (Figure 5B) and shift towards a higher FITC MFI (Figure 5C). Treatment of HLMVECs with CFH induces a significant depolarization of the mitochondrial network. This may partially explain the loss of mitochondria seen in previous experiments (Figure 4).

### 3.6 CFH3+ Induces Activation of the Mitochondrial Permeability Transition Pore Leading to mtDNA Release

**FIGURE 6:**
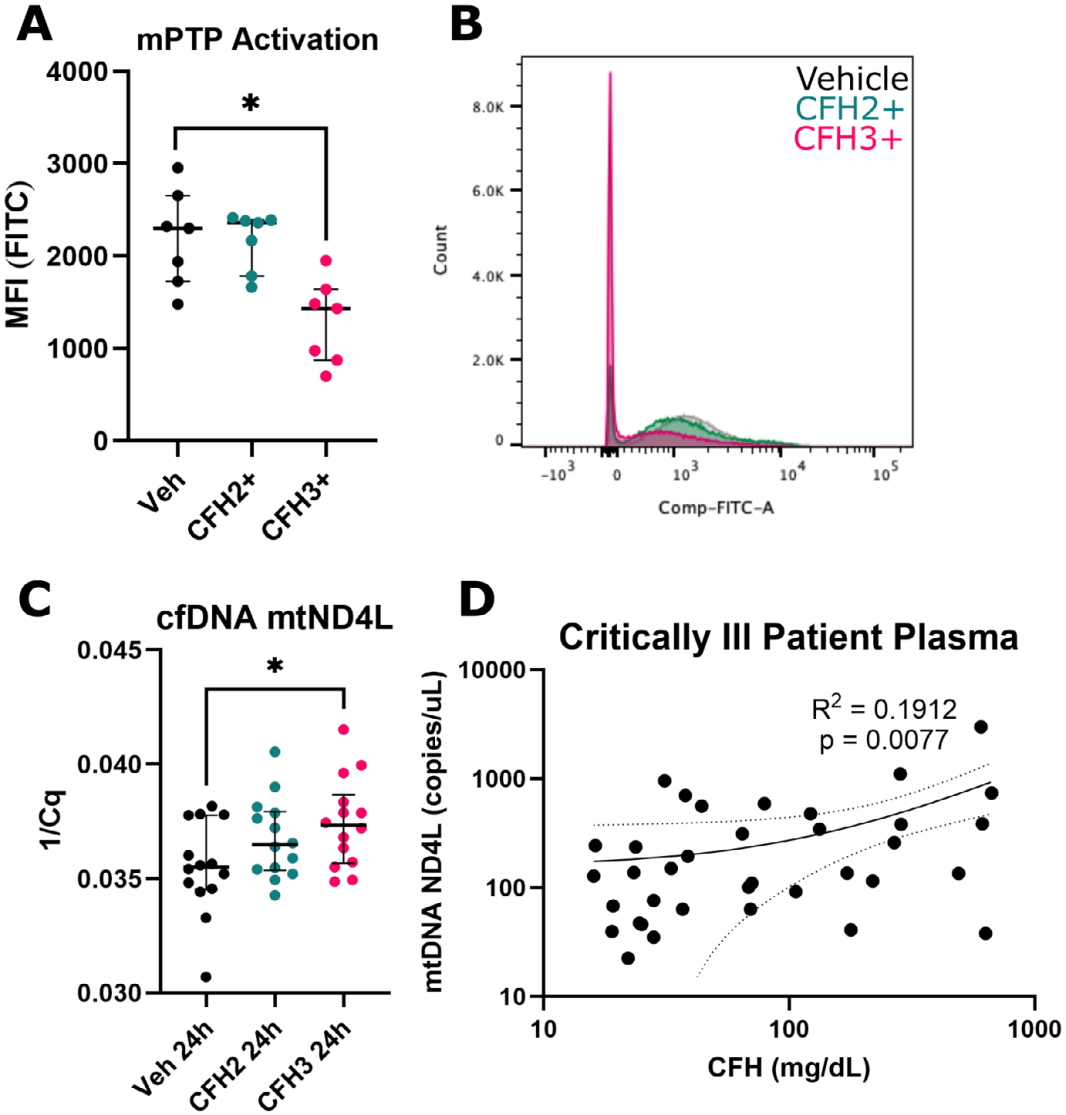
CFH3+ activates the mPTP and induces release of mtDNA. (A) Quantification of HLMVECs stained with calcein‐AM and cobalt chloride measured by MFI. (B) Representative stacked histogram of the FITC fluorescence shift from CFH3+ treatment. (C) Quantification of the change in mtDNA release in the media supernatant by qPCR. (D) Quantification of the circulating mtDNA copy number by ddPCR in patient plasma in association with CFH levels. Probes are specific to the mt‐ND4L gene. (A) *n* = 7 from 3 donors (two male, one female), (C) *n* = 14 from 5 donors (three male, two female), and (D) *n* = 36. * = *p* < 0.05.

Increased ROS and mitochondrial depolarization informed our decision to quantify activation of the mPTP, which is implicated in both conditions [33]. Prolonged activation of the mPTP depolarizes mitochondria and leads to the release of mitochondrial contents into the cytosol. A flow cytometric assay using a combination of Calcein‐AM and cobalt chloride allows for selective staining of mitochondria with inactive mPTP. A significant decrease in the MFI was seen after CFH3+ treatment (1434 [874–1642] vs. 2302 [1729–2654] MFI, *p* = 0.02) when compared to vehicle control (Figure 6A). Activation of the mPTP allows for cobalt chloride to enter the mitochondria and quench the calcein fluorescence. A representative stacked histogram for the FITC channel shows the decrease in MFI that occurs after 6 h of CFH3+ treatment (Figure 6B). We sought to identify if mtDNA, a damage associated molecular pattern, was released after CFH3+ exposure into the extracellular space. Purified DNA from conditioned media was quantified using qPCR with probes specific to mt‐ND4L and expressed as the 1/Cq, so an increase in abundance aligns with an increase in the plotted value. A significant increase in the 1/Cq value was found for CFH3+ treated HLMVECs after 24 h of exposure (Figure 6C). In addition, we sought to identify if circulating mtDNA is associated with CFH concentration in patient plasma. DNA from plasma collected from critically ill patients was purified and the mt‐ND4L copy number was quantified using ddPCR (Table 1). A Spearman correlation with previously measured CFH levels showed a weak association between circulating mtDNA and CFH (*R* ^2^ = 0.1912, *p* = 0.0077) in patient plasma (Figure 6D).

**TABLE 1:** Patient demographics for association between mtDNA and CFH in patient plasma.

Characteristic	*N* = 36 (%)
Sex	
Female	13 (36%)
Male	23 (64%)
White	33 (92%)
Age	55 (44, 71)
APACHE II Score on enrollment	32.5 (27.8, 37.0)^a^
Lived	28 (78%)
Died	8 (22%)
Mechanical ventilation	34 (94%)
Berlin ARDS	16 (44%)
Sepsis on any Day	30 (83%)
Number of PRBC^b^ transfusions on enrollment	
0	27 (75%)
1	1 (2.8%)
2	3 (8.3%)
3	4 (11%)
6	1 (2.8%)

^a^ Median (IQR).

^b^ Packed red blood cell.

## 4 Discussion

In this study, we demonstrate the CFH3+ causes significant changes to the mitochondrial network, including a decrease in area and mass, mitochondrial elongation, superoxide production, and partial depolarization. We found an increase in mtDNA release into the conditioned media potentially mediated by activation of the mPTP by CFH3+. Contrasting these results, which indicate deleterious defects to the mitochondrial network, metabolic analysis showed significant increases to maximal respiration and spare respiratory capacity, while basal respiration remained unchanged.

CFH has several effects on the microvasculature. We previously reported that the treatment of the pulmonary endothelium with CFH results in decreased transendothelial electrical resistance and endothelial activation [17, 18]. These prior studies largely used commercially available CFH3+ without analyzing the effects of CFH2+ and CFH3+ in parallel. Commercial hemoglobin contains impurities that could influence experimental findings [21]. CFH3+ was shown to have a larger impact on activating the endothelium through pro‐inflammatory cytokine production and that the oxidation of CFH was required for endothelial injury [34, 35]. We hypothesized that CFH3+ would induce mitochondrial dysfunction in pulmonary microvascular endothelial cells. The results from the experiments shown here demonstrate a clear difference in mitochondrial defects induced by CFH2+ vs. CFH3+ with only transient decreases in mitochondrial mass and polarization (Figures 4 and 5). This suggests that CFH alone in the native state has limited effects on mitochondrial function and requires oxidation to exert its effects. Our findings agree with previous studies of CFH‐induced endothelial dysfunction and demonstrate that CFH3+ is the primary driver of this dysfunction [17, 19, 36].

Myriad molecular pathways have been implicated in CFH‐mediated toxicity including TLR4, NLRP3, and ROS [14, 15, 19, 24]. Elevated ROS in the endothelium impairs mitochondrial energetics and calcium homeostasis in atherosclerosis [37]. In addition, treatment of alveolar epithelial cells with CFH results in translocation of HO‐1, the heme detoxification enzyme, to the mitochondria and partial mitochondrial depolarization [19]. Our results reveal that multiple mitochondrial defects occur in response to CFH3+ but not CFH2+ treatment. The decrease in mitochondrial mass, area, and polarization supports this hypothesis and is aligned with the previous experiments showing severe barrier disruption and adhesion of leukocytes from CFH exposure [17, 18]. Disruptions in both bulk mitochondria and functional polarized mitochondria pose significant challenges to maintaining oxidative phosphorylation and cellular viability. Without proper metabolism, cells will be unable to perform their vital functions in tissue homeostasis.

Mitochondrial morphology is an important determinant of mitochondrial function [38, 39]. The mitochondrial network is dynamic and becomes fragmented, fused, or hyperfused depending on energy needs and oxidative stress [40]. Fission fragments mitochondria and is important for apoptotic induction and quality control of damaged mitochondria by mitophagy [41, 42]. On the other hand, fusion of mitochondria results in an elongated and connected network of multiple mitochondria to respond to elevated energy needs or to increase electron transport chain (ETC) efficiency [41, 43]. The decrease in roundness and circularity of the mitochondria treated with CFH3+ indicates elongation of the mitochondria and therefore the ability to respond to increased energy needs. The Seahorse data confirm this finding due to the highly significant increase in spare respiratory capacity, a measure of a cell's ability to respond to increased energy needs. Interestingly, the Seahorse assay showed no changes to basal respiration, proton leak, and ATP production, but significant increases in maximal respiration and spare respiratory capacity were seen from CFH3+ treatment. The spare respiratory capacity is a measure of the max respiratory rate of the ETC compared to basal respiration and represents the ability of a cell to respond to increased energy needs or stressful environments [31]. While we hypothesized that fission/fusion proteins would be involved, we did not see any changes in fission/fusion proteins. The absence of changes to basal respiration was surprising but not unexpected. The underlying mechanism of how CFH3+ increases spare respiratory capacity remains unknown [31]. Additional studies evaluating changes to both glycolytic rate and metabolic flux of fatty acids are needed to better understand the metabolic consequences of CFH exposure.

Elevated ROS from CFH is a known mechanism of CFH‐mediated toxicity and here we confirm that CFH3+ increases ROS [15, 19, 44]. Cycling of the iron molecule of heme generates ROS directly and increases oxidative damage [15, 19]. HLMVECs treated with CFH3+ showed elevated cellular ROS and mitochondrial superoxide at the 6 and 24 h timepoints. Attempts to reduce the ROS through antioxidants, such as vitamin C, MitoQ, and acetaminophen, were not effective in ameliorating the increase in ROS production (Figure S1). Although elevated ROS is seen here, the antioxidants added may not be able to alleviate such a high dose of CFH or CFH could be acting indirectly to chronically produce ROS. Elevated ROS led us to look at downstream effectors that are activated by ROS. The mPTP has long been implicated in mediating pathologic mitochondrial dysfunction [33, 45]. The mPTP is a large pore in the mitochondrial inner membrane that facilitates diffusion between the mitochondria and cytosol [33, 46]. ROS, Ca^2+^ ions, and ADP are known activators of the mPTP [33]. mPTP activation was significantly elevated after treatment with CFH3+. We further evaluated whether mtDNA, a molecule that can diffuse through the mPTP, was released from HLMVECs in higher amounts than vehicle or CFH‐treated cells. The increase of −2 to 3 fold demonstrated that activation of the mPTP coincides with the release of mtDNA from CFH3+ treatment. As a clinical correlate, we observed an association between circulating CFH and mtDNA copy number in plasma from critically ill patients. Elevated ROS caused by CFH3+ may be an activator of the mPTP leading to the release of mtDNA from cells.

Our study has strengths and limitations. Studies were done in primary human lung microvascular endothelial cells from multiple donors of both sexes. In addition, we used multiple methods to evaluate many aspects of the endothelial mitochondria from mass and area to activation of the mPTP. However, we did not address the effects of CFH in vivo. Whether these endothelial changes in response to CFH occur in vivo is an unanswered question. The in vitro studies presented here are in‐depth analyses of mitochondrial dynamic changes that are difficult to measure in whole tissue. Second, we identified novel differential effects of CFH 2+ and 3+ in ROS generation and induction of mitochondrial dysfunction, but the underlying mechanisms need to be explored further. A limitation of the study is the relatively low sample size that may obscure more subtle changes in mitochondrial parameters and provides less robust statistical performance. While the mPTP is activated by CFH3+, many other pathways are likely implicated in the complex mitochondrial changes seen here that warrant further investigation.

## 5 Perspectives

The data presented here show that CFH3+ induces mitochondrial dysfunction in the pulmonary microvascular endothelium. This occurs alongside changes in mitochondrial shape and oxidative respiration, and the release of mtDNA from cells. In conditions associated with elevated CFH, mtDNA presents both a novel biomarker and therapeutic target.

## 6 Conclusion

CFH3+ caused significant mitochondrial morphological changes alongside partial depolarization of the network. Mitochondrial maximal respiration was increased from CFH3+ treatment, leading to a significant increase in the spare respiratory capacity. Increased ROS and activation of the mPTP occurred, leading to the release of mtDNA from cells. This study presents a novel mechanism of CFH‐induced endothelial dysfunction that occurs through mitochondrial disruption.

## Consent

Written informed consent was obtained and approved by the Vanderbilt Institutional Review Board, Protocol No. 051065.

## Conflicts of Interest

The authors declare no conflicts of interest.

## Supporting information


Figures S1–S4.

## Data Availability

All data are available upon reasonable request to the corresponding author.
