# Supplementary material for: Oxidized Cell‐Free Hemoglobin Induces Mitochondrial Dysfunction by Activation of the Mitochondrial Permeability Transition Pore in the Pulmonary Microvasculature
Source: Microcirculation. 2025 May 20;32(4):e70012. doi: 10.1111/micc.70012 (PMC12093041; doi:10.1111/micc.70012)
Supplement: Supplementary file 1 — Figures S1–S4. [file MICC-32-e70012-s001.docx]

Electronic Supplemental Materials


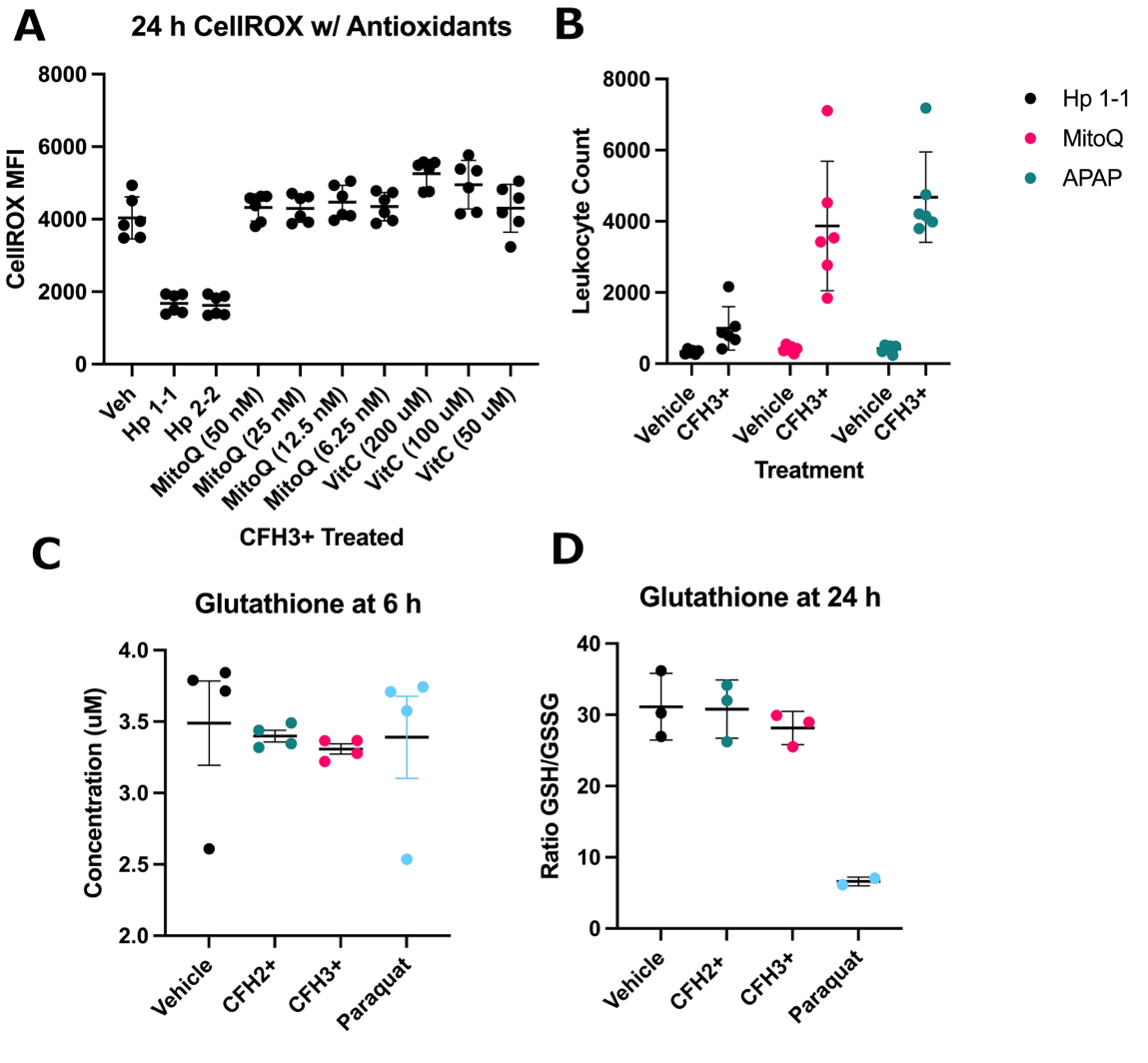


**Supplemental Figure 1: Antioxidants do not ameliorate CFH3+ dysfunction.** **(A)** CellROX assay using increasing doses of MitoQ and Vitamin C in comparison to Hp positive control. **(B)** Leukocyte adhesion assay using MitoQ and acetaminophen (APAP) in comparison to Hp for reducing leukocyte adhesion from CFH3+. **(C)** Glutathione concentrations at 6 h. **(D)** Ratio of reduced to oxidized glutathione at 24 h. (A,B) n = 6, (C) n =4, and (D) n = 2-3).


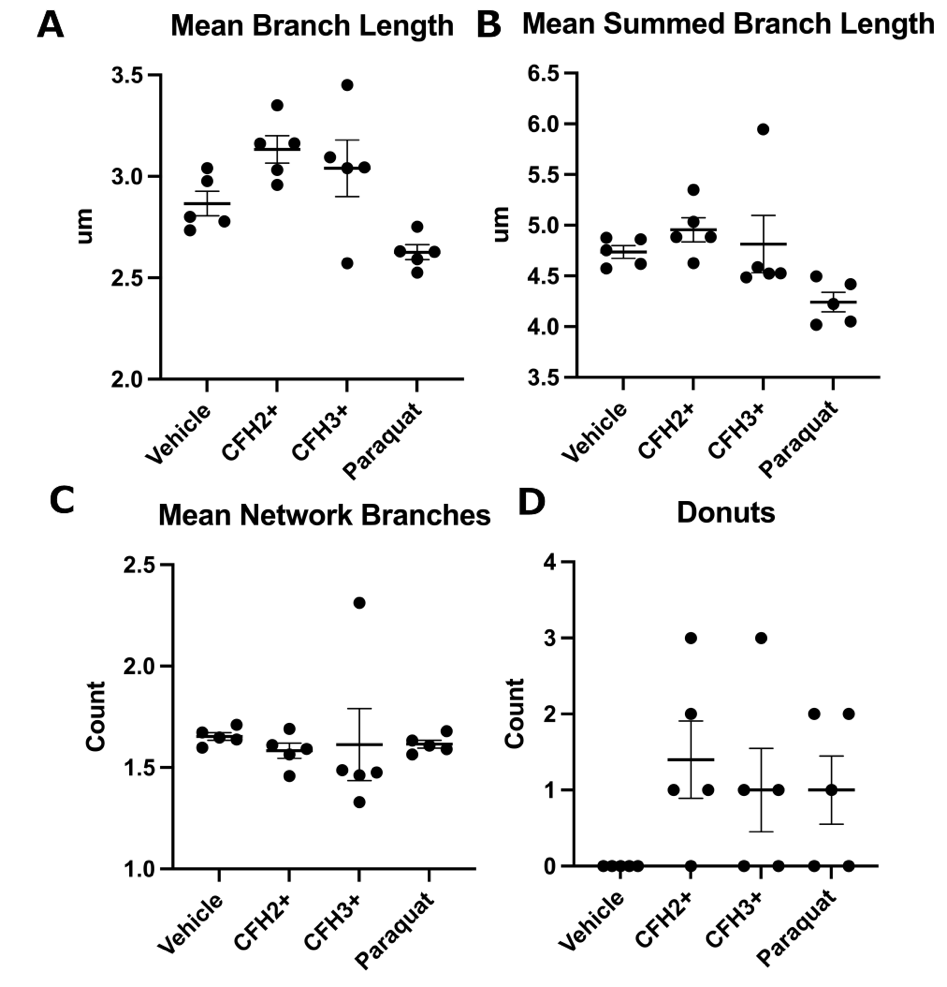


**Supplemental Figure 2: Mitochondrial network parameters not changed by treatment.** Quantification of MiNA outputs of **(A)** mean branch length, **(B)** mean summed branch length, **(C)** mean network branches, and **(D)** donuts. n = 5.


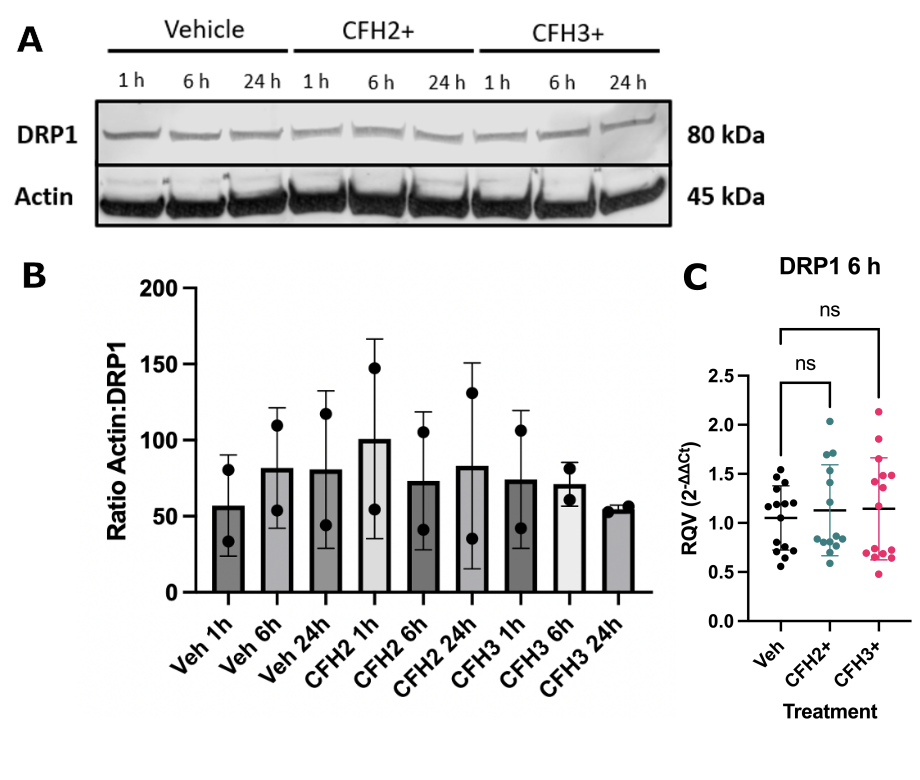


**Supplemental Figure 3: DRP1 expression not affected by CFH.** **(A)** Representative blot of DRP1 expression from HLMVECs and **(B)** quantification of ratio of Actin:DRP1. **(C)** mRNA expression of DRP1 by qPCR from HLMVECs. (A,B) n = 2, (C) n = 14-15.


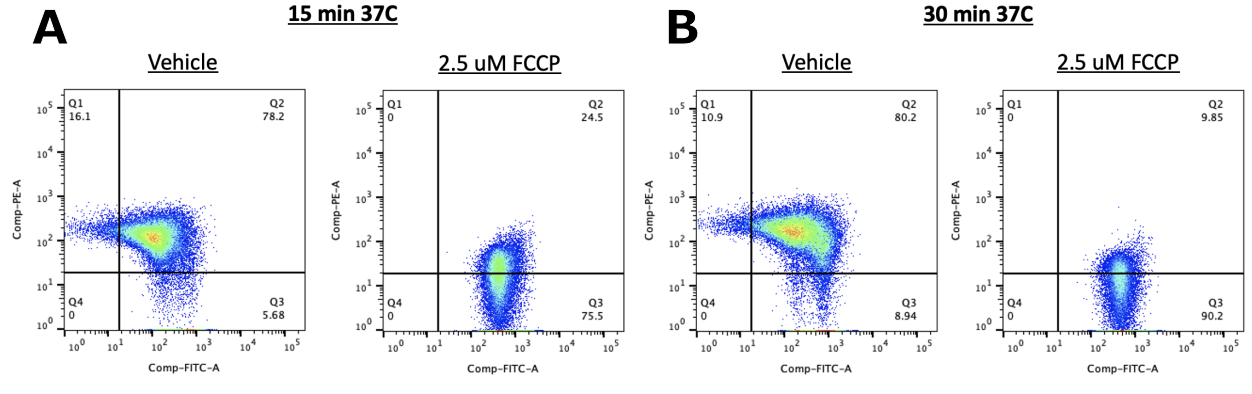


**Supplemental Figure 4: Optimization of FCCP treatment for JC10 assay.** Treatment with FCCP at 2.5 uM with incubation times of **(A)** 15 min and **(B)** 30 min. Quantified by flow cytometry using the FITC and PE channels.
